# Supplementary material for: Spin Hall and Edelstein effects in chiral non-collinear altermagnets
Source: Nat Commun. 2025 Sep 26;16:8529. doi: 10.1038/s41467-025-64271-8 (PMC12475269; doi:10.1038/s41467-025-64271-8)
Supplement: Supplementary file 1 — Supplementary Information [file 41467_2025_64271_MOESM1_ESM.pdf]

Supplementary Information for "Spin Hall and Edelstein Effects in Chiral Non-collinear Altermagnets"

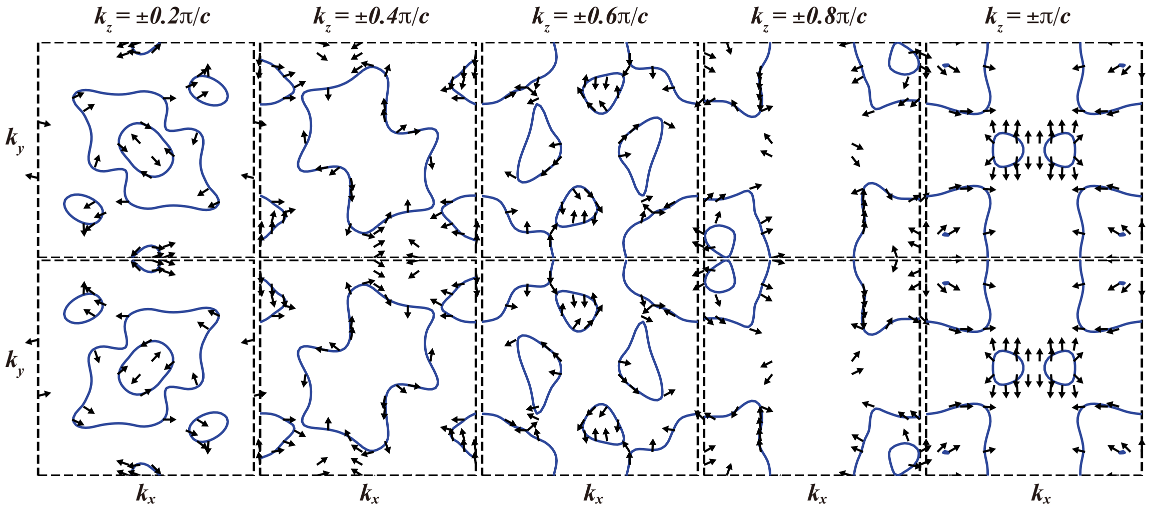

Supplementary Figure 1. Fermi surfaces and spin texture components $\boldsymbol{s}_{x/y}$ at different k_z planes computed by using the Wannier model of Mn_3IrSi. The spin-orbit coupling is included. The actual Fermi energy of Mn_3IrSi was used.

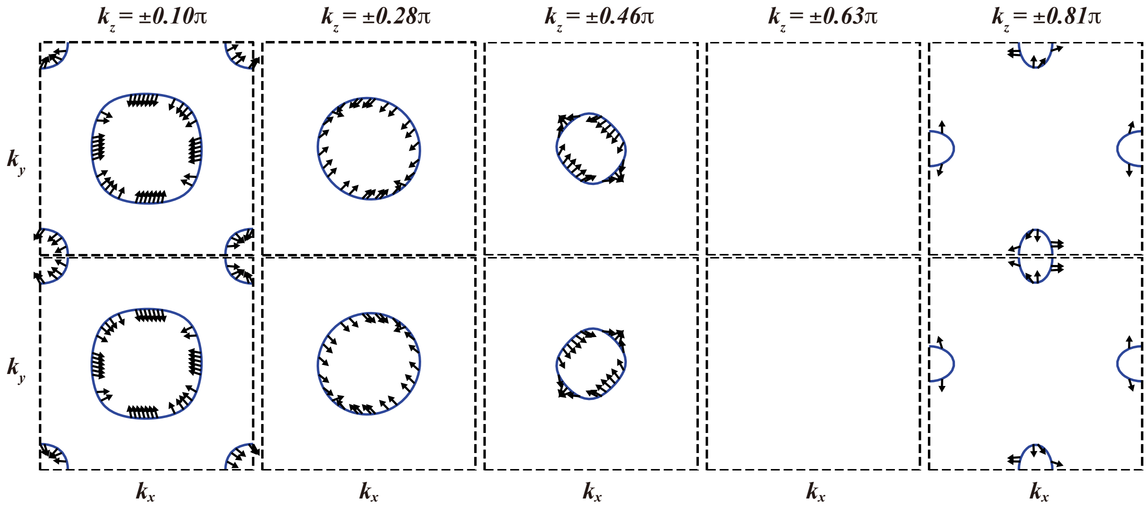

Supplementary Figure 2. Fermi surfaces and spin texture components $\boldsymbol{s}_{x/y}$ at different k_z planes from the model. The Fermi energy is taken as $E = E_f + 0.08$ eV. The spin-orbit coupling is included.

Supplementary Discussion 1. CONDUCTIVITY CALCULATIONS

In the main text, the general Kubo formula is provided, and the corresponding spin currents are calculated. Here, we discuss the charge conductivity results from both the toy model and Wannier functions.

a. Charge conductivity tensor Following the same expression in the main text:

$$\chi^{I} = -\frac{e\hbar}{\pi NV}\sum_{\boldsymbol{k},m,n}\frac{\Gamma^2\,\mathrm{Re}(\,\langle n\boldsymbol{k}|\hat{A}|m\boldsymbol{k}\rangle\ \langle m\boldsymbol{k}|\hat{\boldsymbol{v}}\cdot\boldsymbol{E}|n\boldsymbol{k}\rangle)}{[(E_f-\epsilon_{n\boldsymbol{k}})^2+\Gamma^2][(E_f-\epsilon_{m\boldsymbol{k}})^2+\Gamma^2]},$$
$$\chi^{II} = -\frac{2\hbar e}{NV}\sum_{\boldsymbol{k},n\neq m}^{\substack{n=occ\\m=unocc}}\frac{\mathrm{Im}(\,\langle n\boldsymbol{k}|\hat{A}|m\boldsymbol{k}\rangle\ \langle m\boldsymbol{k}|\hat{\boldsymbol{v}}\cdot\boldsymbol{E}|n\boldsymbol{k}\rangle)}{(\epsilon_{n\boldsymbol{k}}-\epsilon_{m\boldsymbol{k}})^2}. \tag{1}$$

We take $\hat{A}=\hat{v}$ as the charge response under an external electric field. Next, we briefly derive the relation between Berry curvature (Ω) and χ^{II} (σ^{II}).

$$\begin{aligned}\sigma^{II}_{ij} &= -\frac{2\hbar e}{NV}\sum_{\boldsymbol{k},n\neq m}^{\substack{n=occ\\m=unocc}}\frac{\mathrm{Im}(\,\langle n\boldsymbol{k}|\hat{v}_i|m\boldsymbol{k}\rangle\ \langle m\boldsymbol{k}|\hat{v}_j|n\boldsymbol{k}\rangle)}{(\epsilon_{n\boldsymbol{k}}-\epsilon_{m\boldsymbol{k}})^2}\\ &= -\frac{2\hbar e}{NV}\sum_{\boldsymbol{k},n\neq m}^{\substack{n=occ\\m=unocc}} f^{ij}_{nm}(\boldsymbol{k}),\end{aligned} \tag{2}$$

we review the equation of Ω:

$$\begin{aligned}\Omega^{n}_{ij}(\boldsymbol{k}) &= -\sum_{n\neq m}\frac{\mathrm{Im}(\,\langle n\boldsymbol{k}|\hat{v}_i|m\boldsymbol{k}\rangle\ \langle m\boldsymbol{k}|\hat{v}_j|n\boldsymbol{k}\rangle-\ \langle n\boldsymbol{k}|\hat{v}_j|m\boldsymbol{k}\rangle\ \langle m\boldsymbol{k}|\hat{v}_i|n\boldsymbol{k}\rangle)}{(\epsilon_{n\boldsymbol{k}}-\epsilon_{m\boldsymbol{k}})^2}\\ &= \sum_{n\neq m} f^{ij}_{nm}(\boldsymbol{k}) - f^{ji}_{nm}(\boldsymbol{k}).\end{aligned} \tag{3}$$

However, for the Hermitian property of velocity operators (${\hat{v}_i}^{\dagger}=\hat{v}_i$), we have:

$$\begin{aligned}f^{ij}_{nm}(\boldsymbol{k}) &= \frac{\mathrm{Im}(\,\langle n\boldsymbol{k}|\hat{v}_i|m\boldsymbol{k}\rangle\ \langle m\boldsymbol{k}|\hat{v}_j|n\boldsymbol{k}\rangle)}{(\epsilon_{n\boldsymbol{k}}-\epsilon_{m\boldsymbol{k}})^2}\\ &= -\frac{\mathrm{Im}(\,\langle n\boldsymbol{k}|\hat{v}_i|m\boldsymbol{k}\rangle^*\ \langle m\boldsymbol{k}|\hat{v}_j|n\boldsymbol{k}\rangle^*)}{(\epsilon_{n\boldsymbol{k}}-\epsilon_{m\boldsymbol{k}})^2}\\ &= -\frac{\mathrm{Im}(\,\langle m\boldsymbol{k}|\hat{v}_i|n\boldsymbol{k}\rangle\ \langle n\boldsymbol{k}|\hat{v}_j|m\boldsymbol{k}\rangle)}{(\epsilon_{n\boldsymbol{k}}-\epsilon_{m\boldsymbol{k}})^2}\\ &= -f^{ji}_{nm}(\boldsymbol{k}) = f^{ij}_{mn}(\boldsymbol{k}).\end{aligned} \tag{4}$$

So we further derive the simplified expression for $\Omega^{n}_{ij}(\boldsymbol{k})=\sum_{n\neq m}2f^{ij}_{nm}(\boldsymbol{k})$, and the relation in the charge conductivity tensor: $\sigma^{II}_{ij}=-\sigma^{II}_{ji}$. Also, the similar expression between $\sigma^{II}_{ij}(\boldsymbol{k})$ and $\Omega^{n}_{ij}(\boldsymbol{k})$ suggests that the charge conductivity results have qualitatively identical properties to Berry curvature.

b. Extrinsic spin Hall and charge conductivity of Mn_3IrSi Following the analysis above, zero net Berry curvature is expected in Mn_3IrSi, whose compensated magnetization is constrained by symmetry. Thus, the intrinsic charge conductivity tensor is zero ($\sigma_{ij}^{II} = 0$). In Supplementary Fig. 3, the extrinsic charge conductivity (σ^I) and SHE are appended. The extrinsic SHE shares the same tensor form as the intrinsic SHE in the main text: $\sigma_{jk}^i = \epsilon_{ijk}\sigma_0 + |\epsilon_{ijk}|\sigma_1$, where ϵ_{ijk} is the Levi-Civita symbol. The identical formality between $\chi^{I/II}$ is due to the absence of anti-unitary symmetry in Mn_3IrSi. For charge conductivity, there is only one independent parameter: $\sigma_{ij}^I = (1 - |\epsilon_{ij}|)\sigma^c$. We also evaluate the spin Hall angle defined as $\frac{e}{\hbar}\frac{\sigma_{jk}^i}{\sigma_{kk}}$, and the results at the Fermi level are: 0.22%, −4.55% for σ_{yz}^x and σ_{zy}^x, respectively. The non-angle-dependent spin Hall angle, defined as $\frac{e}{\hbar}\frac{\sigma_0}{\sigma_{kk}}$, is −2.16%. The spin Hall angle and spin Hall conductivity are also comparable to other non-collinear antiferromagnets [1].

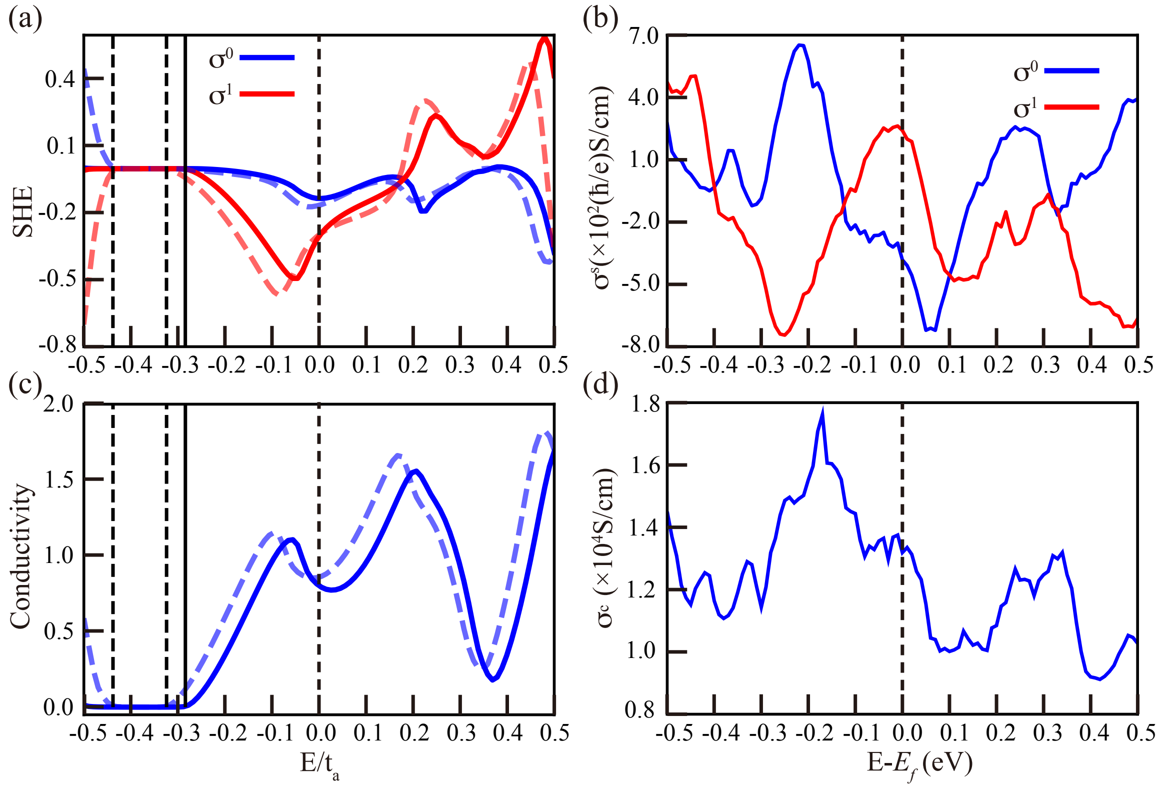

Supplementary Figure 3. The calculated SHE from the extrinsic part (a,b) and charge conductivity (c,d) from both the toy model (a,c) and projected Wannier functions (b,d). The light dashed line in (a) and (c) is from the toy model without SOC. All Γ values take 10^{-2}.

Supplementary Discussion 2. CALCULATIONS OF MAGNETIC EXCHANGES IN MN$_3$IRSI

We assume that the leading magnetic exchanges are short-range and restrict our analysis to those with $d_{\mathsf{Mn..Mn}} \leq 5$ Å. There are 12 such exchanges (see Supplementary Table 1); the three shortest, J_1, J_2, and J_3, together form a trillium lattice. To estimate these exchanges, we use the generalized gradient approximation (GGA) and calculate the total energies of various collinear magnetic arrangements. These energies are mapped onto a classical $S = \frac{3}{2}$ Heisenberg model. The $P1$ cell, metrically equivalent to the cubic unit cell of Mn_3IrSi, allows for 2048 different collinear magnetic configurations. Assuming the magnetism is fully described by the twelve short-range exchanges (Supplementary Table 1), only 184 of these configurations are inequivalent. Three configurations failed to converge; the remaining 181 total energies were used to parameterize the $S = \frac{3}{2}$ Heisenberg model. Parameterization is done by a least-squares solution of the redundant linear problem, which gives the following exchanges (note that some of the exchanges cannot be resolved without doubling the cell):

$$\begin{aligned} J_1 &= 14.0(5)\text{ meV} & J_4 + J_{10} &= -1.2(3)\text{ meV} \\ J_2 + J_8 &= 23.0(4)\text{ meV} & J_5 + J_{11} + J_{12} &= -1.9(5)\text{ meV} \\ J_3 + J_9 &= 11.8(4)\text{ meV} & J_6 &= 1.2(3)\text{ meV} \\ & & J_7 &= 1.4(3)\text{ meV} \end{aligned} \tag{5}$$

Supplementary Table 1. Short-tange ($d_{\mathsf{Mn..Mn}} \leq 5$ Å) magnetic exchanges in the crystal structure of Mn_3IrSi. Interatomic distances correspond to the 5 K structure from Ref. 2.

exchange	$d_{\mathsf{Mn..Mn}}$, Å	multiplicity	exchange	$d_{\mathsf{Mn..Mn}}$, Å	multiplicity
J_1	2.6888	24	J_7	4.5483	24
J_2	2.7206	24	J_8	4.5677	24
J_3	2.7658	24	J_9	4.6360	24
J_4	3.3450	24	J_{10}	4.6962	24
J_5	3.9885	24	J_{11}	4.6976	24
J_6	4.4663	24	J_{12}	4.8354	24

The error bars and the fit quality (Supplementary Fig. 4) are reasonable for a metallic material, in which the lengths of the Mn moments—averaged over all Mn sites and all computed configurations—yield 3.22(10) μ_B. The good agreement with the GGA energies *a posteriori* confirms our assumption that longer-range exchanges are small. Following this logic, we can further assume $J_2 \gg |J_9|$ and $J_3 \gg |J_{11}|$. This leaves us with a distorted windmill lattice model comprising three antiferromagnetic exchanges[3], J_1, J_2, and J_3 (see Supplementary Fig. 7 for the crystalline environments of these exchanges), whose relative strengths are approximately $1:2:1$. All further exchanges are at least about five times smaller than these leading terms.

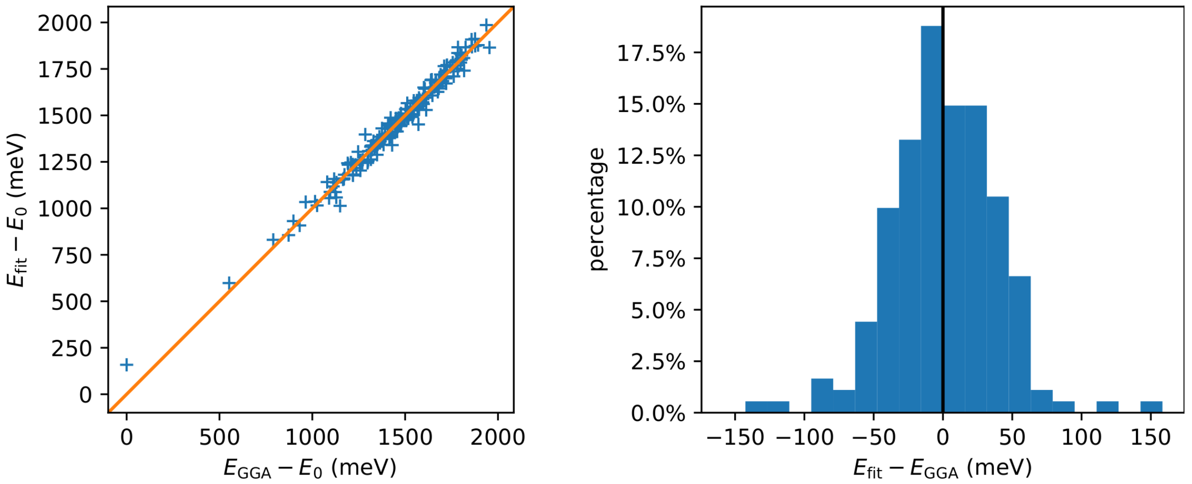

Supplementary Figure 4. Least-squares solution [Supplementary Eq. 5] of the classical $S = \frac{3}{2}$ Heisenberg model parameterized with GGA energies for different collinear magnetic configurations of Mn_3IrSi. Left: differences between the total energies E_{fit} obtained from the least-squares solution and the corresponding GGA total energies E_{GGA} (the diagonal, which marks zero difference, is a guide to the eye). Right: histogram of the differences $E_{fit} - E_{GGA}$.

Supplementary Discussion 3. MONTE CARLO SIMULATIONS OF THE HEISENBERG MODEL AND COMPARISON WITH THE EXPERIMENTS

We compute the magnetic susceptibility of the classical J_1-J_2-J_3 Heisenberg model by performing classical Monte Carlo simulations on $4\times4\times4$ finite lattices (each unit cell comprises 12 spins) with periodic boundary conditions, assuming $J_1:J_2:J_3 = 1:2:1$ (the minimal model of Mn_3IrSi), and compare it to the regular trillium lattice ($J_1 = J_2 = J_3$). Both curves (Supplementary Fig. 5) show a distinct kink that indicates long-range magnetic ordering. The transition temperature of $0.14\bar{J}$ ($0.16\bar{J}$) for $J_1 : J_2 : J_3 = 1 : 2 : 1$ ($J_1 = J_2 = J_3$) corresponds to $S^2\bar{J}/k_B \simeq 60\,\mathrm{K}$, which severely underestimates the experimental ordering temperature of $\sim$225 K. This underestimation is due to the absence of itinerant electrons in the Heisenberg model.

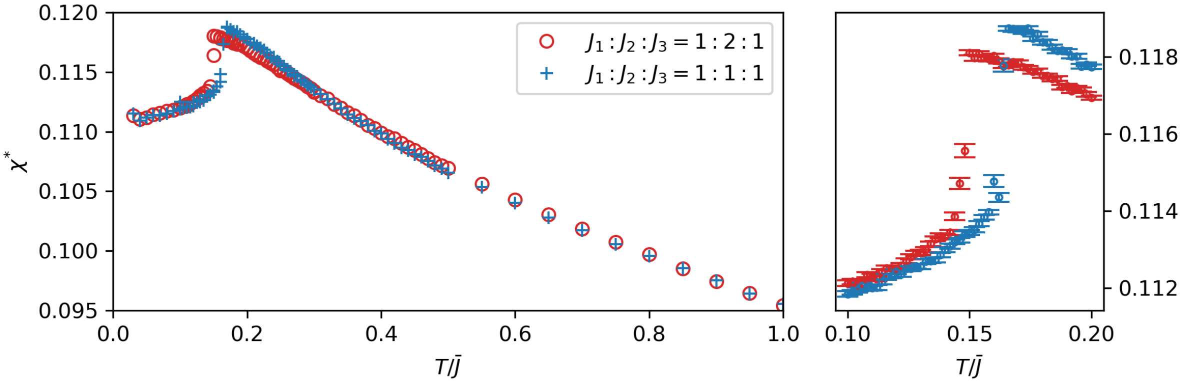

Supplementary Figure 5. Magnetic susceptibility of the classical distorted windmill lattice Heisenberg model as a function of temperature.

The structure of the ordered ground state can be analyzed by inspecting the spin correlations corresponding to J_1, J_2, and J_3 bonds. In Supplementary Fig. 6, we plot their evolution with temperature. At low temperatures, all three curves approach $\langle \mathbf{S}_i \cdot \mathbf{S}_j \rangle = -0.5$, which corresponds to a 120° angle between the magnetic moments. This directly proves that the non-collinear magnetic structure in Mn_3IrSi arises primarily from magnetic frustration, which is induced by antiferromagnetic isotropic interactions between localized moments on a trillium lattice.

After confirming that our spin model features a non-collinear magnetic ground state, we are now in a position to compare our results with experiment. To this end, we inspect the angles between the magnetic moments in the ordered state. The magnetic structure

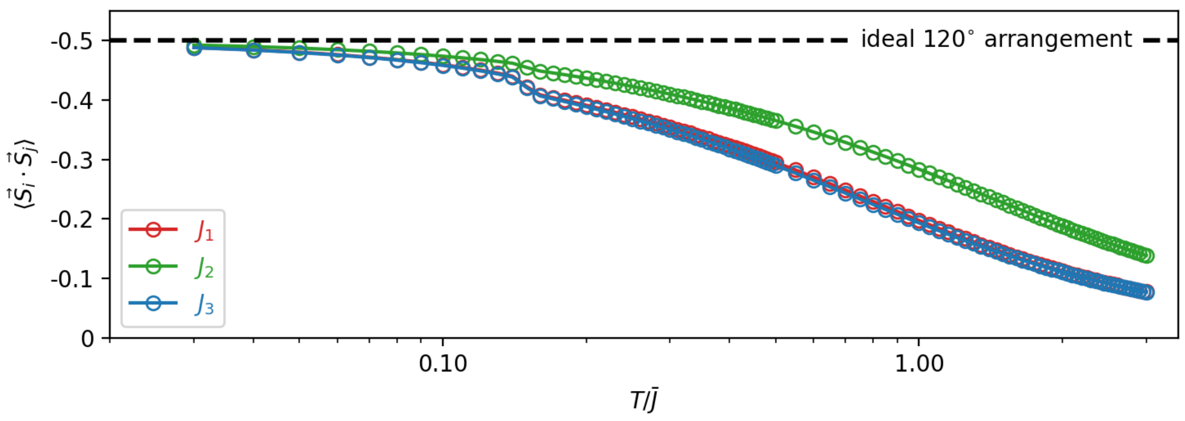

Supplementary Figure 6. Spin correlations in the classical distorted windmill lattice Heisenberg model as a function of temperature.

of Mn_3IrSi has been measured at 5 and 10 K, as reported in Refs. 2 and 4, respectively. Note that slight differences in the atomic coordinates affect the interatomic separations: while the strongest exchange J_2 is the second shortest Mn..Mn separation (2.721 Å) in Ref. 2, it corresponds to the shortest separation (2.678 Å) in Ref. 4. To avoid confusion, we summarize the relevant crystallographic data in Supplementary Table 2. We further note that despite the similarity of the bond lengths and the associated difficulties in the naming convention, the local crystalline environments of the spin triangles are quite different (Supplementary Fig. 7): the triangle formed by J_1 is enclosed in a bipyramid whose apices are Si and Mn atoms, while triangles formed by J_2 and J_3 are bases of pyramids with, respectively, an Ir or Si atom at the apex. The strongest exchange J_2 is thus associated with a triangle having an adjacent Ir atom (Mn_3Ir pyramids).

Finally, in Supplementary Table 3, we compare the angles between the magnetic moments in both magnetic structures. In the structure from Ref. 4, the angle between the moments on J_2 bonds is notably close to 120°. Angles on J_1 and J_3 are significantly smaller, which agrees very well with our estimates. In the magnetic structure from Ref. 2, this tendency is less pronounced: angles on J_2 and J_3 are similar and somewhat smaller than 120°. More conclusive analysis is impeded by sizable standard deviations (see Supplementary Table 3), which amount to several degrees.

Supplementary Table 2. Comparison of structural parameters from Refs. 2 and 4.

parameter	T. Eriksson *et al.*, Phys. Rev. B **69**, 054422 (2004)	A. E. Hall *et al.*, Phys. Rev. Mater. **7**, 114402 (2023)
T, K	10.	5.
a, Å	6.48790	6.49081
Mn x/a	0.11950	0.1187
Mn y/b	0.20310	0.2074
Mn z/c	0.45730	0.4544
$d(J_1)$, Å	2.699	2.689
$d(J_2)$, Å	2.678	2.721
$d(J_3)$, Å	2.796	2.766

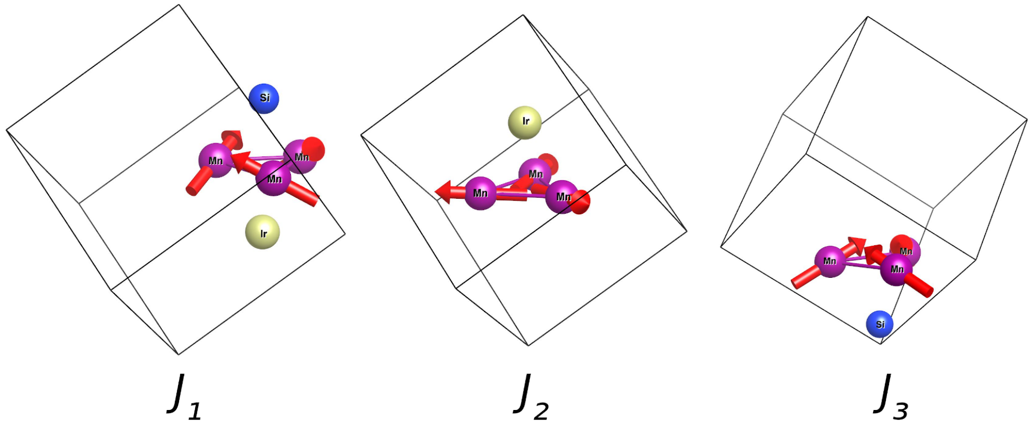

Supplementary Figure 7. Crystalline environments of spin triangles formed by J_1 (left), J_2 (centre), and J_3 (right) bonds in the crystal structure of Mn_3IrSi.

Supplementary Table 3. Angles (in °) between local magnetic moments on Mn atoms. Standard deviations computed from that of $[m_x, m_y, m_z]$ are given in brackets.

bond	$\angle(m_{\mathrm{Mn}_i}, m_{\mathrm{Mn}_j})$ Eriksson *et al.*, Phys. Rev. B 69, 054422 (2004)	Hall *et al.*, Phys. Rev. Mater. 7, 114402 (2023)
J_1	100(3)	104(2)
J_2	119.4(5)	111(3)
J_3	103(2)	111(4)

Supplementary Discussion 4. WANNIER PROJECTION AND ELECTRONIC PROPERTIES WITHOUT SOC OR MAGNETIZATION

As shown in Supplementary Fig. 8, the projected Wannier functions have well-fitted band structure around Fermi level, besides some entangled crossings. The degeneracies at high symmetry points are still consistent between band structures in Supplementary Fig. 8. After symmetrization, negligible changes happen in the band structure, and this Wannier functions based tight-binding model is used for the conductivity calculations.

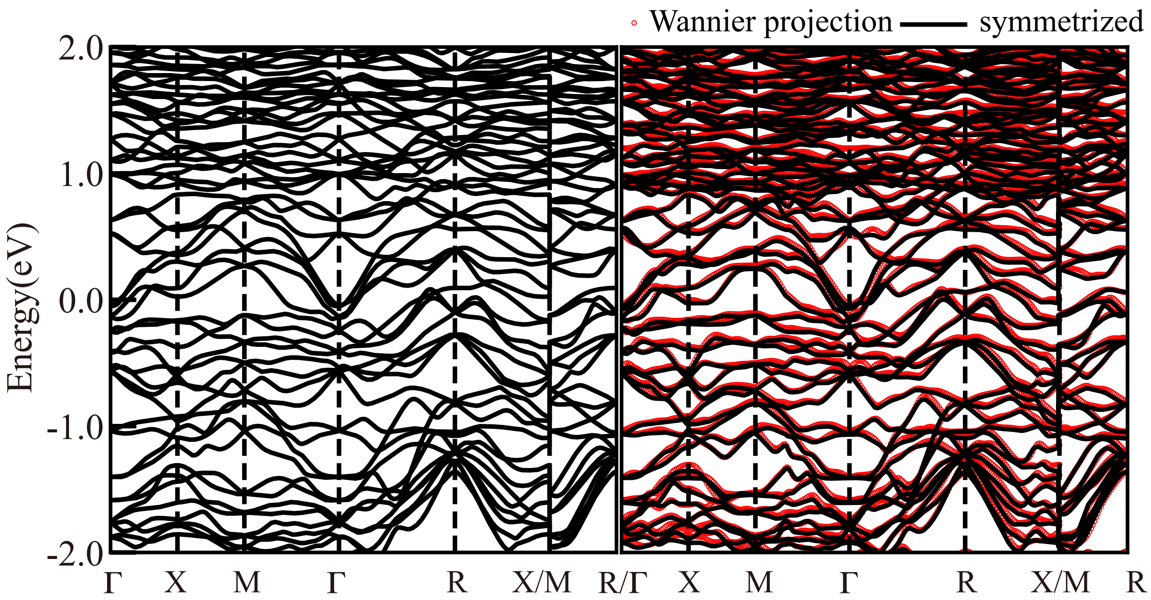

Supplementary Figure 8. Band structures of Mn_3IrSi from first-principles calculations (left panel) and Wannier projection (right panel). The well-fitted region is from $E_f - 2.0 \sim E_f + 1.0$ eV.

a. Effects of SOC and magnetization on band structures As the crystal of Mn_3IrSi is chiral already, the magnetization is manually turned off in our first-principles calculation shown in Supplementary Fig. 9 left panel. In the nonmagnetic case, even higher degeneracies happen at high symmetry points: all states are four-fold degenerated at R. With chiral non-collinear magnetization, the high degenerated states are split and still preserved some multifold degeneracies as shown in Supplementary Fig. 9 right panel. It also is noticed that the SOC influence on band structure is relative small that only varies the velocity.

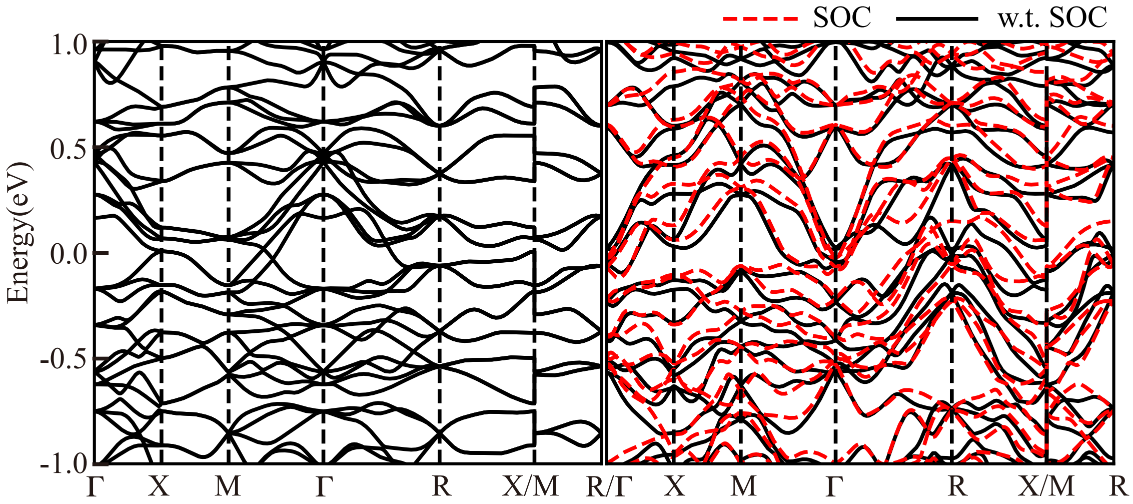

Supplementary Figure 9. Mn_3IrSi band structures with zero local magnetization (left panel), with and without SOC (right panel). In the nonmagnetization calculation, the SOC is also turned off.

b. Spin texture in the presence of SOC Due to the isomorphic relation between the spin space group and the magnetic space group, hedgehog and quadrupole spin textures are expected in Mn_3IrSi without SOC, as shown in the main text. Due to the negligible effect of SOC, both the band structure and spin texture do not show visible differences. As shown in Supplementary Fig. 10, distributions similar to those without SOC of $s_{x/y}$ and s_z at the $k_z = 0$ plane are presented, which are hedgehog and quadrupole components, respectively. The SOC effect in Mn_3IrSi is reflected in the Fermi velocity changes and the different number of bands contributing to the Fermi surfaces.

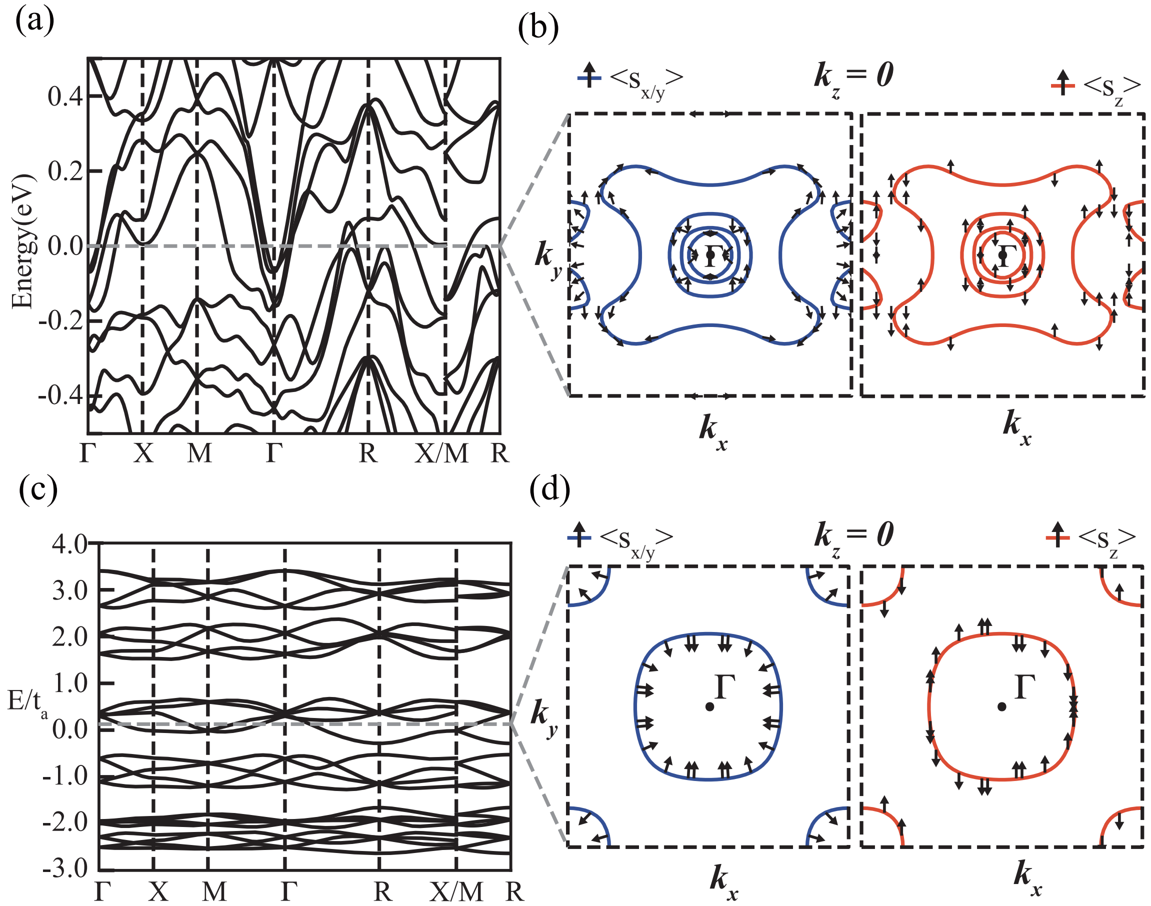

Supplementary Figure 10. Mn_3IrSi band structures (a,c) and spin texture (b,d) with SOC from both the toy model (lower panel) and first-principles calculation (upper panel). The spin texture is taken from the Fermi level and $E = E_f + 0.08$ eV for first-principles calculations and the toy model. Different spin components are shown in (b) and (d) left and right panels, respectively.

c. SHE and Edelstein effect without SOC In the main text, we show the SHE and Edelstein effect with SOC, as this represents the realistic situation. In the previous discussion, we demonstrated the negligible effect of SOC on the band structure and spin texture. Here, we present the simulation results for the SHE and Edelstein effect without SOC in Fig. 11. The Wannier functions are well fitted to the band structure, as shown in Fig. 11a, and the SHE and Edelstein effect are calculated using the same method as in the main text. Both effects are consistent around the Fermi level, with the same order of magnitude, and their trends vary with energy.

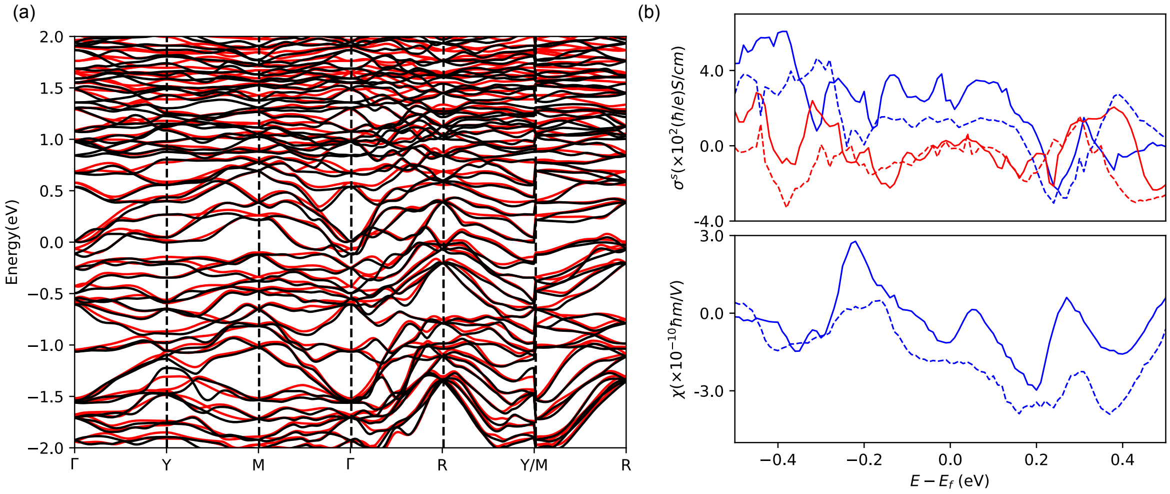

Supplementary Figure 11. (a) Band structure without SOC from DFT calculations (red dots) and projected Wannier functions (black solid lines). (b) Spin Hall (upper panel) and Edelstein (lower panel) effect conductivity tensor elements versus energy around E_f. The red and blue lines represent σ^z_{xy} and σ^x_{xz} in the SHE, respectively. The dashed and solid lines correspond to calculations with full SOC and without SOC, respectively.

[1] J. Železný, Y. Zhang, C. Felser, and B. Yan, Spin-Polarized Current in Noncollinear Antiferromagnets, Phys. Rev. Lett. **119**, 1 (2017), arXiv:1702.00295.

[2] A. E. Hall, P. Manuel, D. D. Khalyavin, F. Orlandi, D. A. Mayoh, L.-J. Chang, Y.-S. Chen, D. G. C. Jonas, M. R. Lees, and G. Balakrishnan, Comparative study of the magnetism in Mn_3RhGe and related compound Mn_3IrSi, Phys. Rev. Mater. **7**, 114402 (2023).

[3] S. V. Isakov, J. M. Hopkinson, and H.-Y. Kee, Fate of partial order on trillium and distorted windmill lattices, Phys. Rev. B **78**, 014404 (2008).

[4] T. Eriksson, R. Lizárraga, S. Felton, L. Bergqvist, Y. Andersson, P. Nordblad, and O. Eriksson, Crystal and magnetic structure of Mn_3IrSi, Phys. Rev. B **69**, 054422 (2004).
